# Tabletop Board Game Elements and Gamification Interventions for Health Behavior Change: Realist Review and Proposal of a Game Design Framework

**DOI:** 10.2196/23302

**Published:** 2021-03-31

**Authors:** Daniel S Epstein, Adam Zemski, Joanne Enticott, Christopher Barton

**Affiliations:** 1 Department of General Practice Monash University Notting Hill Australia; 2 Department of Mathematics Moreton Bay College Brisbane Australia; 3 Monash Centre for Health Research and Implementation Monash University Clayton Australia

**Keywords:** behavior change, games, serious games, board games, behavior interventions, health interventions, health games, game design, tabletop games

## Abstract

**Background:**

Games, when used as interventional tools, can influence behavior change by incentivizing, reinforcing, educating, providing feedback loops, prompting, persuading, or providing meaning, fun, and community. However, not all game elements will appeal to all consumers equally, and different elements might work for different people and in different contexts.

**Objective:**

The aim of this study was to conduct a realist review of tabletop games targeting behavior change and to propose a framework for designing effective behavior change games.

**Methods:**

A realist review was conducted to inform program theory in the development of tabletop games for health behavior change. The context, mechanisms used to change behavior, and outcomes of included studies were reviewed through a realist lens.

**Results:**

Thirty-one papers met the eligibility criteria and were included in the review. Several design methods were identified that enhanced the efficacy of the games to change behavior. These included design by local teams, pilot testing, clearly defined targets of behavior change, conscious attention to all aspects of game design, including game mechanics, dynamics, aesthetics, and the elicitation of emotions. Delivery with other mediums, leveraging behavioral insights, prior training for delivery, and repeated play were also important. Some design elements that were found to reduce efficacy included limited replayability or lack of fun for immersive engagement.

**Conclusions:**

Game designers need to consider all aspects of the context and the mechanisms to achieve the desired behavior change outcomes. Careful design thinking should include consideration of the game mechanics, dynamics, aesthetics, emotions, and contexts of the game and the players. People who know the players and the contexts well should design the games or have significant input. Testing in real-world settings is likely to lead to better outcomes. Careful selection and purposeful design of the behavior change mechanisms at play is essential. Fun and enjoyment of the player should be considered, as without engagement, there will be no desired intervention effect.

## Introduction

### Games as a Tool

Games—activities that one engages in for amusement or fun—have an inherent ability to elicit our interest, engagement, and motivation more than static educational material without implicit rules, objectives, and pursuits [1]. Games can leverage the underlying psychology of rewards, social norms, mastery, autonomy, and pursuit of meaning to achieve desired choices and behaviors among players [2,3]. Gamification describes the purposeful design and application of game-like elements into nongame environments. Although gamification is a broad term, the core principle is taking design elements from games or play to influence choices and behavior [4]. For a game to capture one’s attention and change behavior, it must be carefully designed with a clear goal and consider numerous approaches through multiple lenses [5]. Without a thoughtful design process, gamifying something can render it ineffective or annoying, thereby potentially deterring the desired behavior or promoting undesired outcomes such as cheating or stealing, and in extreme cases, being dangerous or unethical [6,7].

### Tabletop, Card, and Physical Games

In modern times, physical, card, and tabletop games may be considered unsophisticated or outdated and are often overlooked when gamification interventions are considered in favor of contemporary alternatives such as digital or video-based products. However, given that tabletop games are cheaper to produce, arguably easier to design, while promoting an inclusive and social aspect to the gaming experience, they remain a viable alternative for gamification for health behavior change interventions [8]. The universe of game design is vast. The abovementioned reasons are examples of how each aspect of game design can be considered in function toward achieving a desired behavior or goal. Despite the wide scope for application of gamification as a tool, there is minimal literature available to guide its use in the development of behavior change interventions. Overall, there is a lack of contextual understanding of game mechanics and program theories underlying gamification as a tool for behavior change contexts [9].

### A Realist Perspective

Games as a tool in health care is an example of a complex intervention. This is because games are multi-faceted and dynamic, where individual agency and context can change behavior outcomes significantly. Given this, it is reasonable to presume that the same intervention would not work uniformly in different contexts [10]. Realist reviews operate within the lens of realist philosophy and allow a deeper, more nuanced understanding of intervention outcomes than standard systematic reviews. Employing the realist philosophy in the review process, we can unpack an intervention, thereby identifying what underlying mechanisms cause what outcomes, under what conditions, and in what contexts [11] providing explanation, rather than judgement, about how an intervention works [10,11]. This context-mechanism-outcome (CMO) configuration creates an explanatory model for causation. It relies on the theory of complex interventions [12], which theorizes that complex interventions have the properties of complex systems [13]. This is an innovative use of a realist review, as a game design in a behavior change setting has not been formally reviewed with a realist lens. The aim of this study was to review games and game elements designed to affect health behavior change and to propose a framework and program theory to underpin the game design for health behavior change interventions. This review focuses on the use of tabletop games as a behavior change tool in the context of health.

## Methods

### Framework

This realist review uses the framework for realist synthesis described by Pawson et al [14]. After a preliminary reading of the literature, the emerging central review questions generated were as follows:

1. What are effective game mechanics design elements in behavior change interventions?
2. What are the dynamic and context elements that explain outcomes of games and their use as an intervention?
3. Theory integrity and adjudication: do game element theories work as predicted and fit best?
4. Reality testing: how does the intent of gamification translate into practice?
5. Reviewing the current theory and frameworks of game design, can we propose a framework specific for game design in the behavior change context?

### Formulating the Initial Intervention Theory

First, we developed a preliminary program theory following an initial exploratory review of the literature. This paper’s initial program theory framework is built on the broad aspects of game principles by Robson et al [15] using categories of game mechanics, dynamics, and emotions with the addition of aesthetics from the report of Hunicke et al [16]. There are several proposed game design frameworks in the literature—18 were found in a recent systematic review [17]. We conducted additional focused searches of the literature to identify key program theories, thereby refining the search criteria considering emerging data with additional snowball searching to explore new hypotheses as they emerged [14]. The preliminary program theory is based on the hypothesis that games induce desired behavior change through play and engagement. They are a medium for incentivizing, reinforcing, educating, providing feedback loops, prompting, persuading, or providing meaning, fun, and community. However, not all game elements are appealing to all potential users and contexts. Effective game design considers the desired outcome of the intervention and targets behaviors and motivations of the individual player within the context of the choice-making environment [4]. Simply adding points, badges, and leaderboards without a purposeful design is adding gamification without clear outcomes or desired response [4,8]. The literature highlights that a number of things need to be considered such as how participants interact within the game—with each other and themselves. Game design has 4 distinct properties that govern the game experience and can be manipulated to achieve behavioral outcomes [15].

1. Mechanics: The rules of how the game system works.
2. Dynamics: The ways in which the participants interact in response to the mechanics.
3. Aesthetics: The flavor of how the game looks, the storyline, backstory, artwork, physical appearance, or medium.
4. Emotional aspects: How it makes players feel and relate with themselves, the game, and each other.

#### Mechanics

Mechanics determine the rules of the universe that a game creates and will form the foundations of how participants interact. A game can be designed for cooperation or competition and the mechanics dictate how the participants interact with the game and with each other. Clearly stated objectives and the progression toward goals determine how outcomes are pursued and how feedback through gameplay can occur. Success, failure, rewards, or punishments can be used to reinforce behaviors positively or negatively through operant conditioning and intrinsic or extrinsic motivation [15,18,19].

#### Dynamics

Creativity and repeated play within the rules lead to outcomes between the game, players, observers, and spectators that form the game dynamics. All dynamic player behaviors can be designed purposefully to achieve behavior such as cooperation, competition, behavior loops [20], and habit formation. Even negative behaviors can provide a fun dynamics for entertaining and engaging game play [8] such as cheating, bluffing, conspiring, or even quitting [15]. Roles other than players may also form influential dynamics. Passive observers contribute to social relatedness and spectators influence the atmosphere, community, and popularity, and they impact player behavior within the game [15]. Within dynamics, one also needs to consider whether game elements will result in unwanted second-order behaviors [7], undesired or unethical outcomes, and whether there will be lasting or novelty effects on desired outcomes.

#### Aesthetics

The artwork, setting, physical game components, storyline, and immersive objective form an environment that can induce more engaged players and motivate continued play [5].

#### Emotions

Games that trigger emotional responses can be powerful behavior and learning tools but are challenging to design and more difficult to have heterogeneous control over [5,6]. Some emotions have predictable patterns and can be designed to elicit a sense of achievement, mastery, disappointment, or failure. More subtle emotional outcomes can funnel particular behaviors and can be leveraged through research in psychology and behavioral economics tools such as establishing social norms, endowment effects, scarcity, simplification, chance and probability, framing effects, reducing friction costs, network effects, salience, default states, and cognitive loads [4,5].

### Final Systematic Searching for Primary Studies

A final systematic search was performed in December 2019 (Multimedia Appendix 1). We searched 10 databases relevant to health and behavior interventions, including PubMed, Web of Science, and PsycInfo, without date restrictions. The search strategy was developed with the guidance of a medical subject librarian. The search terms included “board game,” “game,” “serious game,” “tabletop game,” “card game,” or “gamification” and “health” but excluding the terms “virtual,” “screen,” or “video,” and all publications were included in the review. We reviewed titles and abstracts to identify relevant studies. The full text of these papers was then accessed to determine if they met the inclusion or exclusion criteria and assessed for relevance and rigor to test against the initial theory [14].

### Inclusion and Exclusion Criteria

**Figure 1:**
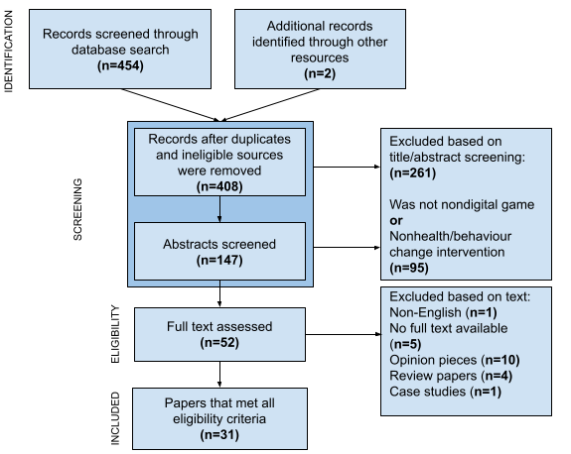
Flow diagram for paper selection.

Studies were included if they used a tabletop game as an intervention and measured a health or behavior change outcome. Studies were excluded if they were digital interventions, did not measure health outcomes, were not English texts, were reviews, opinion pieces, or case studies, or the full text was unavailable upon author request. Papers were initially reviewed by title, keywords, and abstracts for relevance and imported into EndNote reference manager by the first author. Full papers of articles that described behavior change interventions in the context of health were accessed and read in full to determine if they met the inclusion criteria. Papers were excluded if the full text was not available (Figure 1).

### Selecting Evidence

The process of quality appraisal in a realist review is different from that of quality appraisal in a systematic review. Studies are selected based on their relevance toward building and testing theory and their rigor in terms of methodological reliability and description power. A formal methodological quality appraisal tool was not used in this study, but research was individually assessed for rigor.

### Testing Relevance

Studies were expected to have adequate relevance to build the program theory. The quality of the paper was assessed to see if the study addressed the program theory under testing by contributing enough knowledge to comment on the aspects of tabletop games on health outcomes. Any paper that was not directly assessing a tabletop game intervention or not measuring a behavior change outcome was excluded at this point.

### Testing Rigor

The papers were assessed by the reviewers to screen if they were free of bias and were methodologically credible enough to contribute toward the building of the overall program theories. Articles of opinion, review papers, and case studies were excluded, as were non-English texts.

### Extracting Data

A theoretically derived CMO framework was populated to evaluate evidence. Data were extracted and recorded in NVivo software (QSR International) during this process. Information extracted included name of the game, country and setting, method of delivery of intervention, demographics of participants, study design, sample size, comparison group exposure, mechanism of intervention effect, behavior change outcome, and any context-dependent outcomes.

### Analyzing and Synthesizing Findings

To refine the preliminary program theory, we interrogated the literature asking:

1. For *whom* did this basic program theory work and not work, and why?

2. In what *contexts* (C) will this program theory work and not work, and why?

3. What are the main *mechanisms* (M) by which we expect this program theory to work?

4. If this program theory works, what *outcomes* (O) will we see?

This formed the codes used for tabulation, indexing, and linking to the program theory in a CMO configuration for each of the effects and their relevance to strengthen the initial program theory.

The resulting CMO configurations were reviewed by 2 authors (DE and CB) for quality assurance during synthesis. This was achieved by identifying recurring patterns and outcomes in the data, confirming and modifying the reviewers’ understanding of the data, and assessing if the CMO configurations helped inform the hypothesized theory, thereby seeking to both confirm or contradict the findings [21].

## Results

### Literature Review Findings

We identified 454 papers in our initial literature search. Two more papers were identified by hand searching the reference list of included papers. After the removal of duplicates and title screening, 408 records remained. Exclusions in title and abstract screening for applicability (n=261) and exclusions of nondigital games and nonhealth/behavior change interventions (n=95) were made, leaving 52 papers for full-text review. Further papers were excluded where the full text could not be accessed (n=5), or if papers were opinions (n=10), review papers (n=4), or case studies (n=1). One paper was not available in English (n=1). Papers that met all the eligibility criteria (n=31) were included for review (See Figure 1 and Table 1). A description of the included studies can be found in Table 1 and Multimedia Appendix 2. These studies were mainly conducted in Europe (n=13) and North America (n=8) with Africa (n=4), Asia (n=3), and South America (n=2) also having representation. The participants in these studies (20 papers) were mostly younger than 18 years. Several studies had interventions resulting in successful behavior change. The types of successful behavior changes observed included understanding of adolescent smoking [22,23], better nutrition in schools and young people [24-29], recognizing symptoms of delusions and psychosis [30,31], infectious disease education and understanding [32-35], prevention of alcohol abuse [36], chronic disease prevention and management [24,37-40], sexual health practices [32,33,41], and understanding of pregnancy and breastfeeding [42,43]. There was evidence to support the preliminary program theory that identified elements of game design mechanics, dynamics, and aesthetics. Additional consideration was placed onto the context of the participants and research and understanding of the behavior change mechanism components at play (see Table 1 and Table 2).

**Table 1:** Contextual and mechanism factors that enhance efficacy of games in behavior change.

Evidence type/factors	Game design elements described in studies
Strong evidence across many trials	Games developed by local teams rather than designed by distant subject experts [22,27,30,31,40,41,43-45] Games reiteratively developed through pilot testing with target users and context [22,27,29,41-43] Clearly defined behavior-change goals targeted and reflected in game design [22-26,29-31,34,36,37,39,41-43,45-49] Conscious attention to consider game mechanics, dynamics, and aesthetics to increase engagement and target desired behavioral changes [22-32,37,38,41,43,45,46,49] Delivery combined with other mediums or learning modalities [27,28,32,34,35,38,40,46,47] Games that leverage behavioral insights such as social norms, emotive engagement, operant conditioning, or intrinsic motivations [22-26,30,31,41,43]
Limited evidence from one or few trials	Consideration to training before delivery or play [27,29,31,33,41,44,48,49] Multiple exposures to play [23,34,36,39-41,43,45]
Possible factors worth considering in future research	Increasing time series measurement to understand behavior change extinction effects [24,31,33,37,39,42,44]

**Table 2:** Context and mechanisms leading to positive behavior change outcomes in a realistic game design theory.

Mechanisms	Descriptions in the studies
Aesthetics of fun and play increase engagement and information uptake	The fun and attractiveness of games leads to higher attention and engagement, resulting in a positive mechanism for desired behavior change [23,25,29,36,41,45].
Game/social dynamics set social norms, process signaling	Game dynamics create a microcosm of social norms between players and signaling of appropriate actions, resulting in the desired/designed behavior change [23-26,29,31,36,41].
Game mechanics reinforce rules and actions	The rules of the game create clear boundaries and direction for particular actions and desired behavior [23-25,28-31,36,40,41,48,49].
Clear objective/goals leverage internal motivators	Game objectives set attainable goals and motivate players with a sense of purpose, pursuit, and achievement toward the desired behavior [25-29,31,32,35,36,38,39,42,44,45,48-50].
Rewards, success, and failures leverage external motivators	Consequences leverage external motivators and operant conditioning to achieve desired behavior [23,25,27-29,36,38,40-42,46-48,51]
Challenging repeated play leads to competence, mastery, and expertise	Incremental improvement provides feedback on mastery and expertise leading to repeated desired behavior [23-25,28,38,49].
Spectatorship influences atmosphere, community, and expectations	Being observed or creating community reinforces expectations and social norms of desired behavior [24,25,29,31,34-41,43,45,49]

### Effective Game Mechanic Design Elements in Behavior Change Interventions

Overall, in the design and intervention process, there was strong evidence that games developed by local teams rather than distant experts had better outcomes [22,27,30,31,40,41,43-45]. Games that were pilot tested and whose designs were reiterated with target users resulted in better behavior change outcomes [22,27,29,41-43]. Delivery alongside or as an adjunct to other learning modalities was also successful and supports the theory of complex interventions [27,28,32,34,35,38,40,46,47]. There was also evidence that repeated play and training or practice of the delivery of the ruleset and gameplay assisted the outcome [23,34,36,39-41,43,45]. Games were better received and had greater impact when behavior change goals were clearly defined and targeted through the game mechanics, for example, in Kaledo, a collection mechanic will incentivize the players to collect health food–based tokens to redeem in real-life cafeterias for healthy diets [22-26,29-31,34,36,37,39,41-43,45-49].

### Dynamic and Context Elements That Explain Outcomes of Games

Considering and leveraging the dynamics of how players interact with each other to support or enhance the experience resulted in better outcomes [23-26,29,31,36,41]. Interventions that accounted for the development of social norms, process signaling between players, and tapping external and internal motivators were more successful in their goals [22-26,30,31,41,43]. Games that were more fun and enjoyable to play had better information uptake and engagement [23,25,29,36,41,45]. Furthermore, designing the user context and emotions was seen to have an impact on the experience of play. Being observed through spectatorship or creating a community appeared to reinforce expectations and social norms of the desired actions of players [24,25,29,31,34-41,43,45,49].

### Reality Testing: Translation of the Intent of Gamification Into Practice

Games seem to translate well to practice but some intent of the games can be lost, and a framework needs to consider this. There was evidence demonstrating that games that are not enjoyable have limited replayability or have novelty effects that led to poor engagement and less targeted behavior change [23,34,36,40,41,43-45]. This was also seen in games with poor contextual design where information can be misinterpreted or even lead to unwanted effects (Textbox 1 and Table 3) [35,47,50].

**Factors that reduce the efficacy of games targeted for behavior change.**

**Contextual or mechanism factors**

- Laborious or unattractive game designs for immersive play [35,47,50]
- One-off play or limited replayability [23,34,36,40,41,43-45]
- Simple question-and-answer games sometimes that are not engaging or immersive [33,34,36,40,42,44]

**Table 3:** Theories for games targeting behavior leading to poor behavior change outcomes.

Theories	Behavior change outcome
Not fun or enjoyable leads to low engagement	Unpleasant experiences are unlikely to be engaging or repeated and result in no behavior change
Limited replayability reveals novelty effects	One-off play and wearing off of the game novelty results in lack of repetition and lack of lasting behavior change
Poor contextual design leads to misinterpretation	Missing context dues can lead to poor knowledge translation/misinterpretation and unwanted effects

We refined the preliminary program theory based on the CMO analysis. We propose the following modification of the preliminary program theory in order to help future design of games to achieve health behavior change to include more structure and guidance around these results.

### Proposed Framework for Designing Effective Behavior Change Games

#### Define Goal: Diagnosing the Behavior to Change

Like designing any tool for a task, one should start by considering the desired outcome. Define the behavior to change first. List the barriers to behavior change to target.

#### Create Order With Game Mechanics: Building Rules to Deliver the Desired Outcome

There are several mechanisms that can be considered processes to achieving this, such as leveraging behavioral insights, reward and punishment, or information delivery. The mechanism chosen is the skeleton from which the core rules and game mechanics can be built.

#### Expect Chaos Dynamics: Consider How the Individual, Group, and Context Will React to the Rules

Context of the target players, environment, and other constraints at this stage should be reflected upon to consider the dynamics of the player and game interactions.

#### Make it Fun (Aesthetics): Make it Enjoyable for Higher Engagement

To maximize engagement, the aesthetics of the story, visual values, and physical values of the game are important for the player experience. Games that are not fun will not engage players and lead to minimal behavior change.

#### Pilot: Test and Reiterate the Product

A reiterative piloting and feedback loop should be the final stages of the game design.

#### Measure the Desired (and Undesired) Outcomes

Measure with appropriate scientific methods the behavior change defined. Measure the desired outcome, second-order effects, and undesired outcomes. Consider measuring the short and long game of possible behavior change extinction and novelty effects.

#### Use Other Resources: Use as a Tool in a Toolkit

Delivery alongside other mediums will likely increase the desired changes. Including stakeholders as another resource in this toolkit will likely lead to more effective outcomes.

## Discussion

### Principal Findings

The results of this realist review reveal game elements that contribute to health behavior change. Specifically, when careful consideration is used to define and target the behavior, game designers can enhance the game’s mechanics, dynamics, aesthetics, and elicitation of emotions in a refined process to create the best conditions for successful behavior change. We propose the suggested framework for the game design process that will ensure relevance across different regions, settings, and age groups. Behavior change interventions are complex in their nature due to the ways in which behaviors develop in different contexts for different individuals [12,13]. The framework is built on the broad aspects of game principles by Robson et al [15] using broad categories of game mechanics, dynamics, and emotions with the addition of aesthetics from the report of Hunicke et al [16]. There are several proposed game design frameworks in the literature—18 were found in a recent systematic review [17]. Our review suggests that the game design process is mainly focused on the objective of the game. This includes economic frameworks focused on viability in a marketplace for a game and logic framework to ensure that the game makes sense. The more relevant frameworks for behavior change are those focused on the psychology and participant experience. Many of these subset frameworks focus on digital games [52,53] or psychological aspects of gamification in relation to ethics [19,54]. In contrast, the framework outlined in our review aims to be applicable not only to tabletop or board game designs but also to any game being digitized. The review of Mora et al [17] shows that a framework should be actionable and cover the generic basics and should have human-centered design principles at its center.

Game designers need to consider all aspects of the game experience to achieve the desired behavioral outcomes. First, consideration should begin by defining the target behavior and identifying ways to promote this target behavior through the game. Second, a decision should be made on how the game rules will work to reinforce the desired change (game mechanics). This may include examples such as rewards, punishments, goals, collections, information retention, or physical challenge. Next, reflect on the context of how the players interact with the game and with each other (game dynamics) and if this can be leveraged for the behavior change target. This could include cooperation, negotiation, persuasion, competition, subversion, bargaining, spectatorship, charity, and the pursuit of mastery. Finally, fun and enjoyment of the player should be considered, as without engagement, there will be no desired intervention effect. The aesthetics and emotions of the game should not be forgotten as the experience of play should be pleasant and engaging for the maximum effect. This also includes the role of the spectators, observers, and nonparticipants in the process.

Our evidence synthesis demonstrates that our preliminary program theory was insufficient and that the overall design and delivery process has some impact upon the success of the intervention. Games designed by local designers with context-specific knowledge, in addition to training delivered to users in multiple exposures combined with other mediums appear to be important considerations to achieve target behavior change outcomes [23,34,36,39-41,43,45]. Having interested stakeholders and games without novelty effects and good replayability result in feedback loops for incremental gains toward behavior change [23,34,36,40,41,43-45]. Stakeholders who know the types of players and the contexts well should design the games or have significant input. This is consistent with literature that local deliverers of behavior change interventions are more successful at delivering local needs in context [55]. Moreover, reiteration and pragmatic pilot testing in a real-world setting is likely to lead to better outcomes [56,57].

Games appeared to be effective in all age groups, but most of the literature is on participants younger than 18 years. It could be hypothesized that games are only effective for children and adolescents. However, games should be considered a legitimate engaging behavior change intervention for all ages, if the game design process considers the target audience. There is a trend for serious games to be considered a legitimate intervention for a purpose other than pure entertainment. The literature is expanding across all age groups and multiple industries, including applications in military, government, education, corporate business, and health care [58]. There are a range of potential effects that can be seen with interventional games, including perceptual, cognitive, behavioral, affective, and motivational impacts, knowledge acquisition, content understanding, and learning [59,60].

### Pitfalls in Game Design

Several pitfalls were identified for game designers to avoid in each key area. First, the failure to diagnose a specific behavior to target with the game was a common error in the studies reviewed. Games developers need to define the behavior change first, list the barriers to behavior change to target, and follow a structured design process to decide which elements of game mechanics can be leveraged to target this behavior. This process was best done using local teams who have knowledge of the players and context. Second, the dynamics between how the players will interact with the game and with each other should be considered in the context of play to avoid unwanted effects. Thinking about second-order behaviors and possible unwanted effects could be considered here in the design process. Once the rules and predicted play behavior have been established, the user experience and user interface (or aesthetics) should be considered to enhance fun and engagement. Games in which aesthetics was not given due attention were undesirable or not engaging. Finally, the design process should include pilot testing, wherein behavior change will be assessed carefully and a multi-intervention approach will be considered to maximize the impact of the desired outcome, assuming through realism that no 1 intervention will work for everyone.

### Metagaming

The concept of metagaming can also be considered for advanced game design strategy. Designers can abstract outside of the rules of the game to consider how the player will use the game outside of its intended rules or environment [61]. The concept of metagaming is defined as a player taking action outside the rules or design of a game such as developing higher strategy, cheating, applying outside information, or strategy not intended by the mechanics [62,63]. Designing for metagaming behavior is not usually part of the core mechanics but can be intentionally thought of in the design process to add another engaging layer to the game experience. For example, the game itself could be used as a collectable item for a desired outcome, having the physical world around the game change the way it is played or reward cheating in the core mechanics to improve the likelihood of desired behavior outcomes. Considering the target behavior at the metagaming level can advance the engagement and is an interesting opportunity for behavior change game designers.

### Limitations

First, this review was limited by the small number of published studies using games as an intervention. This limits the potential analysis of the topic. Second, the included studies were of varying degrees of methodological quality. One concern is that only a limited number of papers used in this review showed evidence of behavior change in their interventions. Most studies focused on education and knowledge changes owing to the short intervention period and lack of follow-up. As is common in a realist review, we did not exclude papers based on methodological quality. Finally, working definitions of games and the wording of interventions that could be considered games is likely not consistent. It is possible that this review did not contain all evidences on game-like interventions owing to the varying definition of what “is” a game, for example, setting blood pressure target goals could be considered a game element. However, we performed further hand searching of the reference lists from the included studies to mitigate this limitation. This review is also limited in the framework being applicable only to tabletop game contexts.

### Conclusion

Games can be used as a successful intervention tool for effective behavior change. The ability of games to achieve behavior change is a product of their design—to address a clearly defined problem. Using a realist review methodology, we synthesized evidence from published papers and proposed a framework for game design. This framework outlines the considerations for making a game for a behavior change intervention. Game designers should consider elements of this framework specifically early in the design process to map the context and flow of participants through play, help create the mechanics that govern the rules, consider the dynamics and experiential aspects of playing, and maximize the aesthetics of the play experience. This framework is designed to achieve the best possible interventional results without unintentional outcomes in a realist context, acknowledging that not all interventions will work for all participants in all contexts. Game designers can use this framework to improve the quality of the design process for behavior change interventions. Future work in this field is to apply further realist lens to serious game design in fields outside the context of behavior change or tabletop games or to test this thesis framework in the context of digital game design.

## Appendix

Multimedia Appendix 1 Full search string.

Multimedia Appendix 2 Realist review papers included in the full review.

Multimedia Appendix 3 PRISMA checklist.

## Abbreviations

CMO: context-mechanism-outcome
