# Supplementary material for: Tabletop Board Game Elements and Gamification Interventions for Health Behavior Change: Realist Review and Proposal of a Game Design Framework
Source: JMIR Serious Games. 2021 Mar 31;9(1):e23302. doi: 10.2196/23302 (PMC8047814; doi:10.2196/23302)
Supplement: Multimedia Appendix 1 [file games_v9i1e23302_app1.docx]

**Multimedia Appendix 1.** Full search string.

1. (Board game* or serious game* or tabletop game* or card game* or gamification).mp. [mp=title, abstract, original title, name of substance word, subject heading word, floating sub-heading word, keyword heading word, organism supplementary concept word, protocol supplementary concept word, rare disease supplementary concept word, unique identifier, synonyms]
2. health*.mp. [mp=title, abstract, original title, name of substance word, subject heading word, floating sub- heading word, keyword heading word, organism supplementary concept word, protocol supplementary concept word, rare disease supplementary concept word, unique identifier, synonyms]
3. 3 and 4
4. (educat* or behavi* or knowledge or attitude* or learn*).mp. [mp=title, abstract, original title, name of substance word, subject heading word, floating sub-heading word, keyword heading word, organism supplementary concept word, protocol supplementary concept word, rare disease supplementary concept word, unique identifier, synonyms]
5. 5 and 6
6. 5 not 7
7. (Board game* or serious game* or tabletop game* or card game*).mp. [mp=title, abstract, original title, name of substance word, subject heading word, floating sub-heading word, keyword heading word, organism supplementary concept word, protocol supplementary concept word, rare disease supplementary concept word, unique identifier, synonyms]
8. 4 and 9
9. (virtual or screen or video).mp. [mp=title, abstract, originaltitle, name of substance word, subject heading word,floating sub-heading word, keyword heading word,organism supplementary concept word, protocolsupplementary concept word, rare disease supplementaryconcept word, unique identifier, synonyms]
10. 9 not 11
11. 4 and 12
