# Supplementary material for: Tabletop Board Game Elements and Gamification Interventions for Health Behavior Change: Realist Review and Proposal of a Game Design Framework
Source: JMIR Serious Games. 2021 Mar 31;9(1):e23302. doi: 10.2196/23302 (PMC8047814; doi:10.2196/23302)
Supplement: Multimedia Appendix 2 [file games_v9i1e23302_app2.docx]

**Multimedia Appendix 2.** Realist review papers included in the full review.

| **Source** | **name of game** | **location** | **Delivery** | **age range** | **description of participants** | **sample size (clusters)** | **study design** | **learning mechanics** |
| --- | --- | --- | --- | --- | --- | --- | --- | --- |
| akogun[35] | parasite snakes-and-ladders | Nigeria | through community groups | adult | Rural communities | 1383 | cohort study | information delivery |
| amaro[29] | Kaledo | Italy | school teacher run, 15-30min/week for 24 weeks | 9-19 | Middle School Children | 241 (13) | cluster RCT | Action and consequence; Competition |
| Bartfay[50] | The Lifestyle Game | Canada | teacher led, 2 x 1h sessions, two weeks apart | ~10-12 | Students | 23 | RCT | Question and answer; Competition |
| Burghardt[40] | Multiple | USA | pharmacy student led, one off session | adult | Mostly African American adults, low literacy | 193 | Control trial (non randomised) | Question and answer; Discussion/game, facilitator; Competition |
| Charlier[44] | unnamed First aid game | Belgium | teachers prior game knowledge, 1h session | 13-14 | Middle SES, mostly white students | 120 (4) | Cluster RCT | Question and answer; Competition |
| Crawford[39] | Conversation Map | USA | clinical led small groups, 2h/week for 4 weeks | adult | Adults with Diabetes | 411 | retrospective case-control | Discussion/game facilitator; Collaboration |
| Czuchry[36] | Downward Spiral | USA | self-led group, 1h5min once off play | 18-24 | Mostly female Christian university psychology class | 183 | RCT | Action and consequence; Competition |
| Ezezika[28] | Nutrido | Nigeria | School game club led, unlimited casual play over several months | 13 -17 | Students | 31 | Qualitative focus groups | Punishment and reward, real world integration with healthy food discounts for reward |
| Gilliam[23] | Smokestacks | USA | teacher led session | 4-18 | mostly African American, Metropolitan students | 67 | cohort study | shifting attitudes, normative beliefs, and behavioural intentions through role play |
| Gontijo[63] | Unnamed sexual health game | Brazil | multiple mini stations | adolescents | Students | 58 | mixed methods cohort | Q and A, scenario discussion role play, probability luck based, reward based. |
| Harikiran[48] | 32 warriors | India | research assistants trained by the researchers, 20-minute play time | 12-13 | Lower middle-class students | 45 | cohort study | Question and answer; Discussion/game, facilitator; Competition |
| Khazaal 2006[30] | Micheal's Game | Switzerland, Belgium, France | clinicians with previous CBT training | adults | stable psychotic patients with symptoms | 32 | cohort study | Scenario based changing patient's perspective, normalizing psychotic symptoms, cognitive restructuring techniques aiming to develop alternative explanations to their delusions, reality testing and connecting belief to emotion and behaviour |
| Khazaal 2011[31] | Micheal's Game | Switzerland, Belgium, France | clinicians with 2h game training prior | adults | stable psychotic patients with symptoms | 135 | cohort study | cognitive restructuring. It is guided by the cards instructions which elicit Socratic questioning |
| Khazaal 2013[22] | Pick-Klop | Switzerland | 2 x 1.5h lessons two weeks apart | adults | daily smokers | 240 | RCT | Question and answer; Action and consequence; Competition |
| La Torre 2017[46] | Multiple | Italy | mini stations | 7-9 | middle SES students | 89 | RCT | Fact delivery, question and answer |
| La Torre 2018[49] | Sfumiamo | Italy | oral presentation and game delivered by authors | 9-10 | middle SES students | 67 | RCT | Action and consequence |
| Ladur[43] | Whose Shoes | UK | small focus group, 1-2 hours once off | adults | Married men/fathers from Africa originally | 4 | Qualitative focus groups | Scenario role play of empathy, compassion and critical thinking |
| Lakshman[27] | Top-Grub | UK | teacher run, 1h class over 9 weeks | 10-12 | Students | 1133 (25) | Cluster RCT | Punishment and reward decision making |
| Lennon[34] | Goodbye-to-Dengue | Philippines | teacher run, 35min | 11-14 | Students | 168 | RCT | Question and answer; Competition |
| Maclachlan [33] | Unnamed HIV snakes and ladders game | Malawi | teachers with game training, 1h/week for 4 weeks | 14-19 | Students | 828 | Control trial (non randomised) | Question and answer; Competition |
| Martins[42] | Trilha Família Amamenta [Breastfeeding Family’s Trail] | Brazil | 10min instructions, student run small groups, 50 minutes | 7-10 | Students | 99 (9) | Control trial (non randomised) | (not enough detail) |
| McKay[47] | Hold the Salt | USA | teacher run, 6 sessions over 1 month | sixth graders | Urban, African American students | 78 | Control trial (non randomised) | [Not enough detail |
| Schaeffer[45] | Safety Land | USA | camp leader instructed during session | 5-11 | Summer camp attendees | 274 | cohort study | Situational discussion, Q and A |
| Sen[38] | Kaledo | Turkey | clinician led, 40–60 min at 2-week intervals were performed in 3 months | 9-12 | obese children | 52 | RCT | Collection, reward and punishment |
| Sharps[26] | Unnamed fruit and vegetable game | UK | researcher led, 2 x 30min sessions | 6-11 | Students | 143 | RCT | social norms, Question and answer, competition |
| Van Der Stege[41] | SeCZ TaLK | Netherlands | clinicians with game training led, played several times | adolescents | adolescents with chronic conditions or disabilities | 85 | mixed methods cohort | Destigmatization, role play, Q and A |
| Van Scoy[37] | My Gift of Grace | USA | self-directed with instructions, 60-90 minutes | adult | white, mostly female adults | 68 | cohort study | discussion generation, role play and social inclusion |
| Viggiano 2015[25] | Kaledo | Italy | teacher led, 20-30min/week for 20 weeks | 9-19 | Students | 3110 (20) | Cluster RCT | Collection, reward and punishment |
| Viggiano 2018[24] | Kaledo | Italy | teacher led, 20-30min/week for 20 weeks | 7-11 | Students | 1313 | Cluster RCT | Collection, reward and punishment |
| Wanyama[32] | Make a Positive Start Today | Uganda | clinician led, 20-30 minutes | 24-40 | HIV+ve patients | 180 | RCT | Question and answer; Competition |
